# Blood-Bourne MicroRNA Biomarker Evaluation in Attention-Deficit/Hyperactivity Disorder of Han Chinese Individuals: An Exploratory Study

**DOI:** 10.3389/fpsyt.2018.00227

**Published:** 2018-05-29

**Authors:** Liang-Jen Wang, Sung-Chou Li, Min-Jing Lee, Miao-Chun Chou, Wen-Jiun Chou, Sheng-Yu Lee, Chih-Wei Hsu, Lien-Hung Huang, Ho-Chang Kuo

**Affiliations:** ^1^Department of Child and Adolescent Psychiatry, Kaohsiung Chang Gung Memorial Hospital, Chang Gung University College of Medicine, Kaohsiung, Taiwan; ^2^Department of Chinese Medicine, Chang Gung University, Taoyuan, Taiwan; ^3^Genomics and Proteomics Core Laboratory, Department of Medical Research, Kaohsiung Chang Gung Memorial Hospital, Chang Gung University College of Medicine, Kaohsiung, Taiwan; ^4^Department of Psychiatry, Kaohsiung Veterans General Hospital, Kaohsiung, Taiwan; ^5^Department of Psychiatry, College of Medicine, National Yang-Ming University, Taipei, Taiwan; ^6^Department of Psychiatry, Kaohsiung Chang Gung Memorial Hospital, Chang Gung University College of Medicine, Kaohsiung, Taiwan; ^7^Department of Pediatrics, Kaohsiung Chang Gung Memorial Hospital, Chang Gung University College of Medicine, Kaohsiung, Taiwan; ^8^Kawasaki Disease Center, Kaohsiung Chang Gung Memorial Hospital, Kaohsiung, Taiwan

**Keywords:** ADHD, epigenetic, diagnosis, miRNA, biomarker

## Abstract

**Background:** Attention-deficit/hyperactivity disorder (ADHD) is a highly genetic neurodevelopmental disorder, and its dysregulation of gene expression involves microRNAs (miRNAs). The purpose of this study was to identify potential miRNAs biomarkers and then use these biomarkers to establish a diagnostic panel for ADHD.

**Design and methods:** RNA samples from white blood cells (WBCs) of five ADHD patients and five healthy controls were combined to create one pooled patient library and one control library. We identified 20 candidate miRNAs with the next-generation sequencing (NGS) technique (Illumina). Blood samples were then collected from a Training Set (68 patients and 54 controls) and a Testing Set (20 patients and 20 controls) to identify the expression profiles of these miRNAs with real-time quantitative reverse transcription polymerase chain reaction (qRT-PCR). We used receiver operating characteristic (ROC) curves and the area under the curve (AUC) to evaluate both the specificity and sensitivity of the probability score yielded by the support vector machine (SVM) model.

**Results:** We identified 13 miRNAs as potential ADHD biomarkers. The ΔCt values of these miRNAs in the Training Set were integrated to create a biomarker model using the SVM algorithm, which demonstrated good validity in differentiating ADHD patients from control subjects (sensitivity: 86.8%, specificity: 88.9%, AUC: 0.94, *p* < 0.001). The results of the blind testing showed that 85% of the subjects in the Testing Set were correctly classified using the SVM model alignment (AUC: 0.91, *p* < 0.001). The discriminative validity is not influenced by patients' age or gender, indicating both the robustness and the reliability of the SVM classification model.

**Conclusion:** As measured in peripheral blood, miRNA-based biomarkers can aid in the differentiation of ADHD in clinical settings. Additional studies are needed in the future to clarify the ADHD-associated gene functions and biological mechanisms modulated by miRNAs.

## Introduction

Attention-deficit/hyperactivity disorder (ADHD), a psychiatric disorder commonly found in children and adolescents, is characterized by inattention, hyperactivity, and impulsivity (1). This disorder affects 3–10% of school-age children worldwide (2), and a local prevalence rate of 7.5% was reported in a study of Taiwan (3). Etiology of ADHD may be multifactorial, and neurotransmitters, cytokines and growth factors may be involved in the pathogenesis of this neurodevelopmental disorder (4–6). Early detection and proper diagnosis of ADHD are vital for providing clinicians with the opportunity to intervene and improve patients' functional impairments throughout their life (7). However, the current classification of ADHD is generally based on the observation of behavioral signs and the verbal reports of patients or their family members, rather than a biological basis (8). Since behavioral measures can be subject to both rater bias and reporting bias, the reliability and validity of ADHD diagnosis continues to be heatedly debated (9). Therefore, identifying biomarkers, particularly ones that could be measured *in vivo* with non-invasive methods, may facilitate the differential diagnosis of ADHD, as well as the development of new therapeutic strategies (10).

Genetic factors play an important role in the etiology of ADHD (11), and the heritability for ADHD has been estimated to be around 75–90% (12). The heritability of ADHD is influenced by complex environmental or epigenetic factors (13). Among epigenetic factors, microRNAs (miRNAs) have emerged as possible biomarker candidates due to their involvement in the dysregulation of gene expression (14, 15). miRNAs are small non-coding RNAs that negatively regulate gene expression in human cells (16). More than one-third of human genes have been predicted to be directly targeted by miRNAs (17), which are particularly abundant in the nervous system, where they significantly influence development and likely mediate neuroplasticity (18). The dysregulation of miRNAs may underlie the molecular pathophysiology of various psychiatric and neurological disorders (19–21). Therefore, researchers have become increasingly interested in exploring the role of miRNAs in the pathophysiology of ADHD (22, 23).

Many animal studies have demonstrated that miRNA expression (i.e., rno-let-7d) or miRNA target genes (i.e., Homer 1a) are associated with phenotypes of ADHD animal models (24–28). With regard to human subjects, researchers have investigated the role of gene polymorphisms within miRNAs or miRNA target sites in ADHD pathogenesis (29–36). Some case-control studies eventually focused on differentially expressed miRNAs between individuals with ADHD and healthy control subjects. Significantly lower levels of miRNA 18a-5p, 22-3p, 24-3p, 106b-5p, and 107 and significantly higher levels of miRNA 155a-5p and miRNA let-7d were observed in ADHD patients (37, 38). While the aforementioned studies provided evidence regarding the relationship between the levels of circulating miRNAs and ADHD (37, 38), these miRNAs have yet to be applied as a diagnostic tool for ADHD. Moreover, the selection of candidate miRNAs was based on underlying neurobiology mechanisms (enzymes, carrier molecules, receptors, etc.) that have been identified in the previous literature and in the miRNA database. Therefore, some potential miRNA biomarkers of ADHD that are involved in unknown biological mechanisms may be omitted when not using global screening technology, such as microarray or next-generation sequencing (NGS) (39). NGS is a global screening technique for miRNA expression profile that demonstrates high sensitivity and low background noise (40, 41) and has the potential to discover ADHD markers that have not yet been identified.

Currently, no study has applied the NGS technique to identify miRNA biomarkers in order to assist with the differential diagnosis of ADHD. Although ADHD is a brain-based neurodevelopmental disorder, identifying miRNA expression in brain tissue of living is impossible in clinical settings. Relatively, detecting miRNA expression from peripheral blood is a feasible substituting method. Plasma and white blood cells (WBCs) have been suggested as biomarker sources and to identify circulating plasma-based miRNA biomarkers for neuropsychiatry diseases (42, 43). However, evidence revealed that WBCs are a major contributor to circulating miRNA, and that perturbations in blood cell counts and hemolysis can alter plasma miRNA biomarker levels (44). We propose that combining differentially expressed miRNAs of WBCs can serve as a diagnostic tool for differentiating patients with ADHD from healthy control subjects. The aim of this study is to identify potential miRNA biomarkers through the NGS technique and to assess whether differentially expressed miRNAs can be combined to formulate a reliable and valid diagnostic panel for ADHD.

## Materials and methods

### Study participants

Our research protocol was approved by the Institutional Review Board (IRB) at Chang Gung Memorial Hospital in Taiwan. We obtained written informed consent from the parents or guardians of all the participating children in accordance with the Declaration of Helsinki. This study consisted of eligible patients with ADHD treated in the outpatient Department of Child Psychiatry at Chang Gung Children's Hospital in Taiwan and healthy control children from May 2015 to February 2017. The criteria for ADHD patients included the following: (a) a clinical diagnosis of ADHD by a senior child psychiatrist based on the criteria provided in the Diagnostic and Statistical Manual of Mental Disorders, Fourth Edition, Text Revision (DSM-IV-TR) after structured interviews based on the Chinese version of the Schedule for Affective Disorders and Schizophrenia for School-Age Children, epidemiologic version (K-SADS-E) (45); (b) aged between 6 and 16 years; (c) a Han Chinese ethnic background; and (d) must have never taken any medications to treat their ADHD. We excluded patients with major physical illnesses (such as genetic, metabolic, or infectious conditions) or a history of comorbid major neuropsychiatric diseases (such as intellectual disabilities, autism spectrum disorder, bipolar disorders, major depressive disorders, psychotic disorders, substance use disorders, epilepsy, or severe head trauma).

The healthy control subjects were children without ADHD, who were ethnically Han Chinese and between the ages of 6 and 16 years old within the same catchment area. The participants had to be children without any known major physical illnesses or any of the aforementioned major neuropsychiatric diseases.

**Figure 1:**
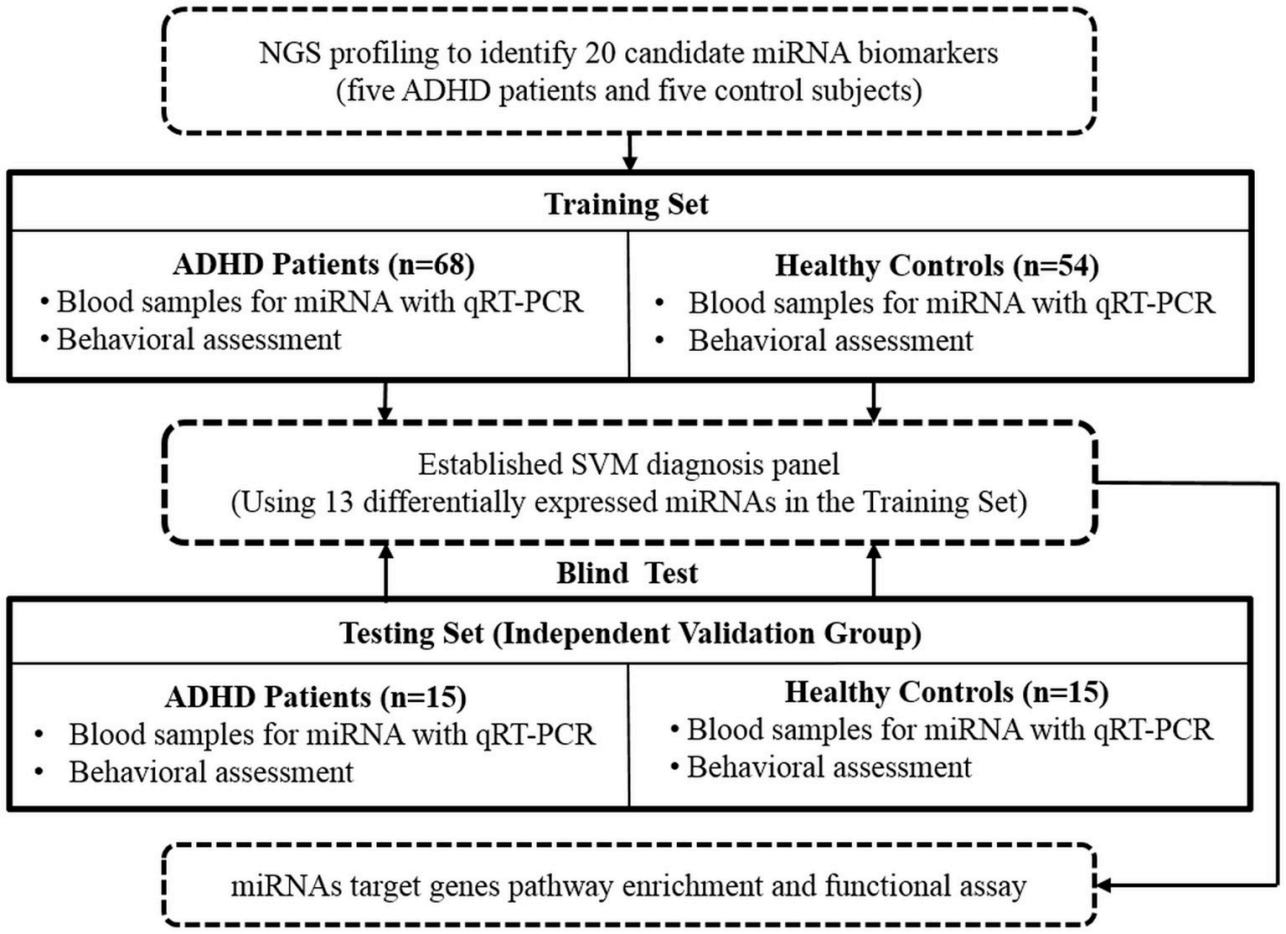
Flow chart of our study design and procedure. NGS, next-generation sequencing; qRT-PCR, real-time quantitative reverse transcription polymerase chain reaction; SVM, support vector machine.

Figure 1 shows the flow chart of our study design and procedure. First, we collected blood samples from five ADHD patients and five healthy control subjects. These samples were then used to explore potential miRNA-based biomarkers of ADHD by using NGS techniques. We subsequently enrolled a Training Set (discovery group: 68 patients with ADHD and 54 healthy controls) to create a miRNA-based diagnostic panel, and finally, a Testing Set (validation group: 20 patients with ADHD and 20 healthy controls) was recruited to verify our findings.

### Collecting clinical blood samples and extracting RNA samples

We collected approximately 5 ml of whole blood from each subject with Vacutainer® Blood Collection Tubes (with EDTA, REF367835, BD, New Jersey, USA). The collected whole blood samples were placed in a centrifuge (3,000 rpm for 10 min) with the red blood cell (RBC) lysis buffer (RBCBioscience) added to remove the RBCs. Next, the RBC-free pellets were collected and processed with the mirVana miRNA isolation kit (Life technology) to extract total RNAs from the WBC. The blood samples in the same batch were processed in parallel. As a result, the whole procedures were finished in 4 h from initial blood drawing to final RNA harvesting. The integrity of the RNAs was determined using Agilent 2100 (Agilent Technologies). We excluded all samples with an RNA integrity number (RIN) less than 8.

### Using NGS to evaluate miRNA profiles

NGS is a highly sensitive technique to globally screen miRNA expression profiles. The RNA samples from the five ADHD patients and five healthy controls were evenly mixed to generate one pooled patient library and one control library, respectively. The two pooled RNA libraries were prepared with TruSeq Small RNA Preparation protocol (Illumina). Then, the prepared amplicon products were examined with high-sensitivity DNA 1000 kit of Bioanalyzer 2100 (Agilent) to confirm successful preparation. Next, the amplicon products were sequenced with a V3 150-cycle sequencing reagent on the MiSeq facility (Illumina) to generate 51-nt single-end reads. The generated NGS data were analyzed with miRSeq tool kit (46) with default parameters to quantify the expressions of human miRNAs (miRBase 21). In summary, there were 9.89 and 9.63 million reads in the control and disease samples, respectively. The raw NGS data was also submitted to NCBI SRA database with the accession number SRP140588.

### Real-time qPCR validation of miRNA

We employed real-time quantitative reverse transcription polymerase chain reaction (qRT-PCR) to determine the expression profiles of the 20 miRNAs in all the samples from the enrolled subjects. The RNA samples were first reverse transcribed into cDNAs with High-Capacity cDNA Reverse Transcription Kit (Applied Biosystems, CA, USA) as the manufacturer's instructions. Then, we further used the TaqMan MicroRNA Transcription Kit (Applied Biosystems, Foster City, CA, USA) to prepare the RNA samples. The sequence and Assay ID for each miRNA kit are available in Supplementary Table 1. Reverse-transcription reactions were carried out on a Veriti 96-well thermal cycler (Applied Biosystems) pursuant to the manufacturer's instructions. The real-time PCR cycling conditions were 95° for 10 min, followed by 40 cycles of 95° for 15 s and 60° for 1 min. We determined miRNA expression abundances using ΔCt values with the small nucleolar RNU44 as the endogenous control.

### Clinical measurements

A senior psychiatrist used the K-SADS-E diagnostic tool (45) to interview all the participants in both the ADHD patient group and the control group. Furthermore, an experienced child psychologist conducted the Wechsler Intelligence Scale for Children–Fourth Edition (WISC-IV) (47) with individual patients in a room designed to reduce variability in testing conditions. The Swanson, Nolan, and Pelham Version IV Scale (SNAP-IV) parent form and SNAP-IV teacher form (48) were completed by the patients' parents and teacher, respectively. Patients were additionally interviewed by a clinician using the ADHD Rating Scale (ADHD-RS) (49).

### Statistical analysis

We analyzed data using the statistical software package SPSS, version 16.0 (SPSS Inc., Chicago, IL, USA) and the MedCalc software Version 15.11.4. Variables were presented as either the mean (standard deviation) or frequency. Two-tailed *p*-values of < 0.05 were considered statistically significant.

We applied the Chi-square test or Fisher's exact test to compare gender distribution between the ADHD patients and the controls. An independent *t*-test or Mann-Whitney U test was utilized to determine the potential difference in age, clinical assessments, and miRNA expressions between the ADHD patients and healthy controls. The Multivariate Analysis of Covariance (MANCOVA) was used to examine whether age, sex, or intelligence quotient functioned as confounding factors in miRNA expression between ADHD patients and controls.

A support vector machine (SVM), a type of machine learning algorithm, can effectively handle binary classification problems and is suitable for disease diagnosis or prognosis applications (50, 51). A library for SVM (LIBSVM) is integrated software for support vector classification, regression, and distribution estimation (52). We used receiver operating characteristic (ROC) curves and the area under the curve (AUC) to evaluate both the specificity and sensitivity of the probability score yielded by the LIBSVM. The optimal diagnostic point of the signature is evaluated at the cut-off values at the probability score of 0.5.

We further performed sensitivity analyses to test the robustness of our results. First, we combined all of the participants in the Training Set and Testing Set and categorized them into a younger group and an older group based on the median age of 108 months. We then used ROC analysis to examine whether the probability scores produced by the SVM model effectively differentiates patients from controls in both age groups. The ROC analysis was also applied to investigate the discriminative validity in boys and girls. Finally, we used Pearson correlation to estimate the relationships between the probability score and ADHD symptoms in all patients.

## Results

### Demographic data

Table 1 summarizes the characteristics of the ADHD patients and the healthy controls. In the Training Set, a total of 68 ADHD patients (mean age 9.1 years, 83.8% males) and 54 healthy control subjects (mean age 10 years, 57.4% males) were recruited. Compared to the control subjects, ADHD patients were more likely to be male (*p* = 0.001) and younger (*p* = 0.047) and to have a lower intelligence quotient (*p* < 0.001), higher inattention scores (*p* < 0.001), and higher hyperactivity/impulsivity scores (*p* < 0.001) as rated by parents, teachers, or clinicians.

**Table 1:** Characteristics of patients with ADHD and healthy controls in the Training Set and the Testing Set.

	**Training set**				**Testing set**			
**Characteristics**	**ADHD (*N* = 68)**	**Controls (*N* = 54)**	**Statistic**	***p*-value**	**ADHD (*N* = 20)**	**Controls (*N* = 20)**	**Statistic**	***p*-value**
Sex			10.448	0.001^*^			0.000	1.000
Male	57 (83.8)	31 (57.4)			14 (70)	14 (70)		
Female	11 (16.2)	23 (42.6)			6 (30)	6 (30)		
Age (years)	9.1 ± 2.2	10.0 ± 2.7	2.012	0.047^*^	8.7 ± 2.2	9.2 ± 2.5	0.666	0.509
FSIQ of the WISC-IV	98.1 ± 9.4	106.4 ± 9.7	4.694	<0.001^*^	101.4 ± 10.9	104.3 ± 14.2	0.726	0.472
**CLINICAL MEASURES**								
SNAP-IV parent form (I)	16.6 ± 5.5	5.6 ± 5.8	10.306	<0.001^*^	16.3 ± 5.4	4.7 ± 5.0	6.860	<0.001^*^
SNAP-IV parent form (H)	15.6 ± 6.1	4.5 ± 5.7	9.832	<0.001^*^	15.3 ± 6.2	4.7 ± 5.7	5.550	<0.001^*^
SNAP-IV teacher form (I)	15.6 ± 5.8	3.9 ± 3.5	12.958	<0.001^*^	14.6 ± 6.5	5.9 ± 6.3	4.237	<0.001^*^
SNAP-IV teacher form (H)	12.6 ± 6.4	2.7 ± 3.1	10.634	<0.001^*^	12.4 ± 8.2	2.8 ± 3.6	4.750	<0.001^*^
ADHD-RS (I)	22.6 ± 5.1	1.3 ± 3.8	26.404	<0.001^*^	23.9 ± 3.6	1.4 ± 3.2	20.979	<0.001^*^
ADHD-RS (H)	23.2 ± 5.3	1.4 ± 4.2	25.079	<0.001^*^	24.1 ± 4.3	1.7 ± 4.3	16.548	<0.001^*^

Data are expressed as N (%) or Mean ± SD; FSIQ, Full Scale Intelligence Quotient; WISC-IV, Wechsler Intelligence Scale for Children–Fourth Edition; I, inattention scores; H, hyperactivity/impulsivity scores;

* *p < 0.05*.

### miRNA profiling with NGS

The miRNA profiling data from the NGS technique is shown in Supplementary Table 2. The NGS analysis identified at least 18 potential miRNA candidates with differential expressions between patients and controls. The criteria for miRNA candidates were defined as: (a) TPM (transcript per million) >1,000 in either the ADHD group or the control group; and (b) Ratios of miRNAs of patients/controls were generally higher than 1.50 or lower than 0.67. Combined with the findings of previous case-control studies (37, 38), miR-18a-5p and let-7d were also considered potential biomarkers. In total, 20 miRNAs conformed to the criteria and could potentially serve as diagnostic biomarkers of ADHD.

### Validation with qPCR

**Figure 2:**
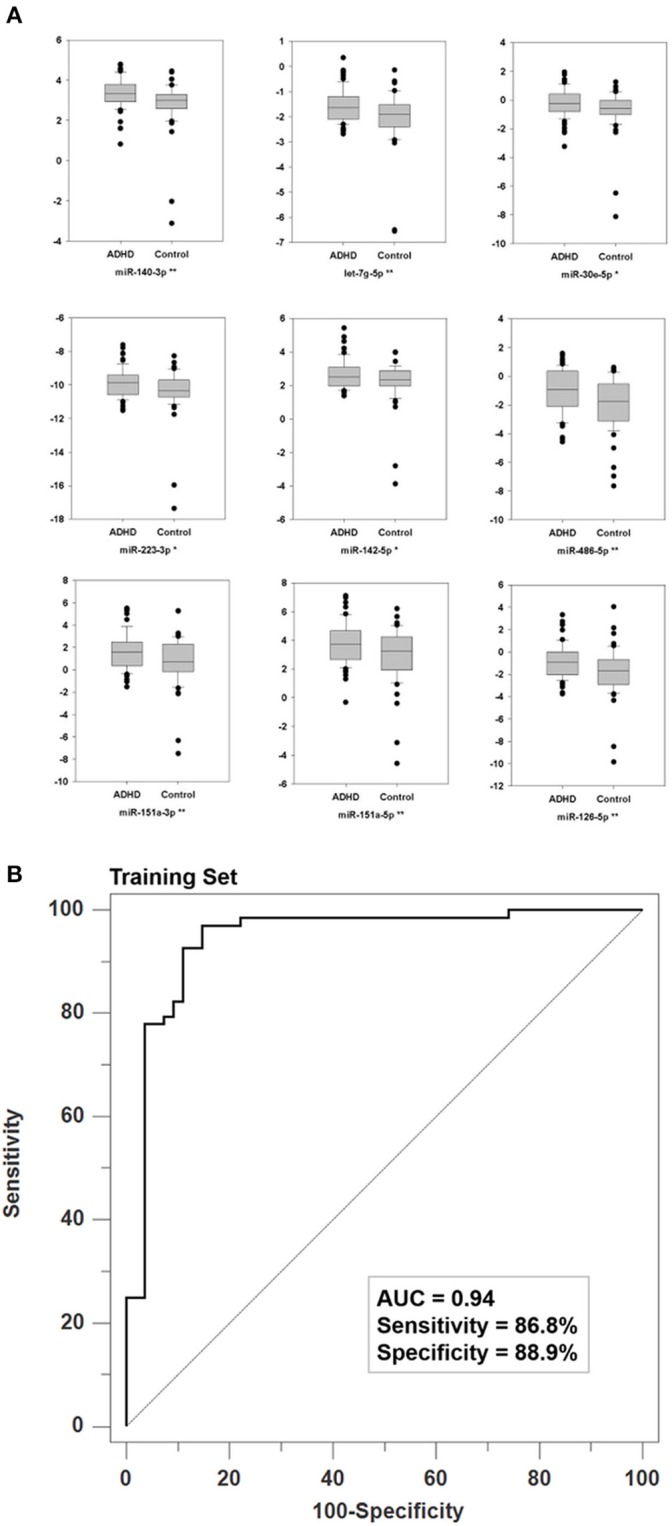
Specific validation of biomarker miRNAs and their discrimination power. **(A)** We used qPCR to validate the expression profiles through NGS in all RNA samples from the Training Set. The Y-axis denotes the ΔCt values with U44 as the internal control. For each miRNA, ^*^ and ^*^^*^ denote *P* < 0.05 and *P* < 0.01, respectively. **(B)** Using the ΔCt values of 13 biomarker miRNAs as vectors (predictors), the Support Vector Machine (SVM) achieved a classification model with an AUC value of 0.94 where the two parameters of gamma and cost were 0.015625 and 256, respectively.

As our NGS data was generated from only one pooled disease library and one pooled normal library, we applied qPCR validation on all samples from the Training Set. By doing so, we found that nine miRNAs were significantly differentially expressed between ADHD patients and healthy controls in the Training Set (*p* < 0.05, Figure 2A). Furthermore, four miRNAs have a *p*-value < 0.1 (Supplementary Figure 1). The raw Ct, ΔCt values and statistical values of the miRNAs examined in the Training Set are presented in Supplementary Table 3. To create a more robust diagnosis model, we selected 13 miRNAs as the candidate biomarkers. The MANCOVA models demonstrated that miRNA expressions were not confounded by age, gender, or intelligence quotient (Table 2). Therefore, we used a combination of the expression levels of these 13 miRNAs to create an ADHD biomarker panel with a SVM algorithm.

**Table 2:** Effects of sex, age, and intelligence quotient on miRNA expression in the Training Set.

**miRNA**	**Sex**		**Age**		**Intelligence quotient**	
	***F***	***p*-value**	***F***	***p*-value**	***F***	***p*-value**
miR-140-3p	0.005	0.943	0.001	0.981	0.149	0.700
miR-27a-3p	0.054	0.817	0.000	0.991	0.004	0.948
miR-101-3p	0.002	0.961	0.612	0.436	0.641	0.425
miR-150-5p	0.760	0.385	0.150	0.699	0.681	0.411
let-7g-5p	0.066	0.798	0.031	0.861	0.013	0.911
miR-30e-5p	0.000	0.994	0.045	0.832	0.171	0.680
miR-223-3p	0.385	0.536	0.000	0.992	0.146	0.703
miR-142-5p	0.026	0.871	0.093	0.761	0.020	0.887
miR-92a-3p	0.001	0.975	0.023	0.879	0.191	0.663
miR-486-5p	0.195	0.660	0.065	0.800	0.109	0.742
miR-151a-3p	0.371	0.544	2.474	0.119	0.053	0.818
miR-151a-5p	0.294	0.589	0.148	0.701	0.193	0.661
miR-126-5p	0.083	0.774	0.275	0.601	0.116	0.734

*Data are expressed using the Multivariate Analysis of Covariance, controlling for the diagnostic group (ADHD vs. controls)*.

### The ADHD diagnosis model

We used the ΔCt values of the 13 biomarker miRNAs from the training set as SVM vectors to create an ADHD biomarker panel. To obtain the best discriminative validity, we first used five-fold cross validations to determine the optimal parameters: gamma = 0.015625 and cost = 256. The SVM classification model produced a probability score that could differentiate ADHD patients from healthy controls. Using a default probability cutoff of 0.5, the biomarker panel has a sensitivity of 86.8% and a specificity of 88.9%, leading to an AUC value of 0.94 (*p* < 0.001; Figure 2B).

To examine the strength of this biomarker panel, an independent cohort that included 20 ADHD patients and 20 healthy control subject was recruited as the Testing Set to conduct a blind test. No significant differences were found in age, gender, or intelligence quotient between ADHD patients and controls (Table 1). We determined the miRNA ΔCt of the 40 subjects in the Testing Set. After SVM model alignment, 18 of the 20 ADHD samples were correctly classified as ADHD, and 16 of the 20 healthy control samples were classified as not having ADHD, demonstrating a sensitivity of 90%, a specificity of 80%, and an accuracy of 85% (AUC: 0.91, *p* < 0.001).

### Sensitivity analyses

**Figure 3:**
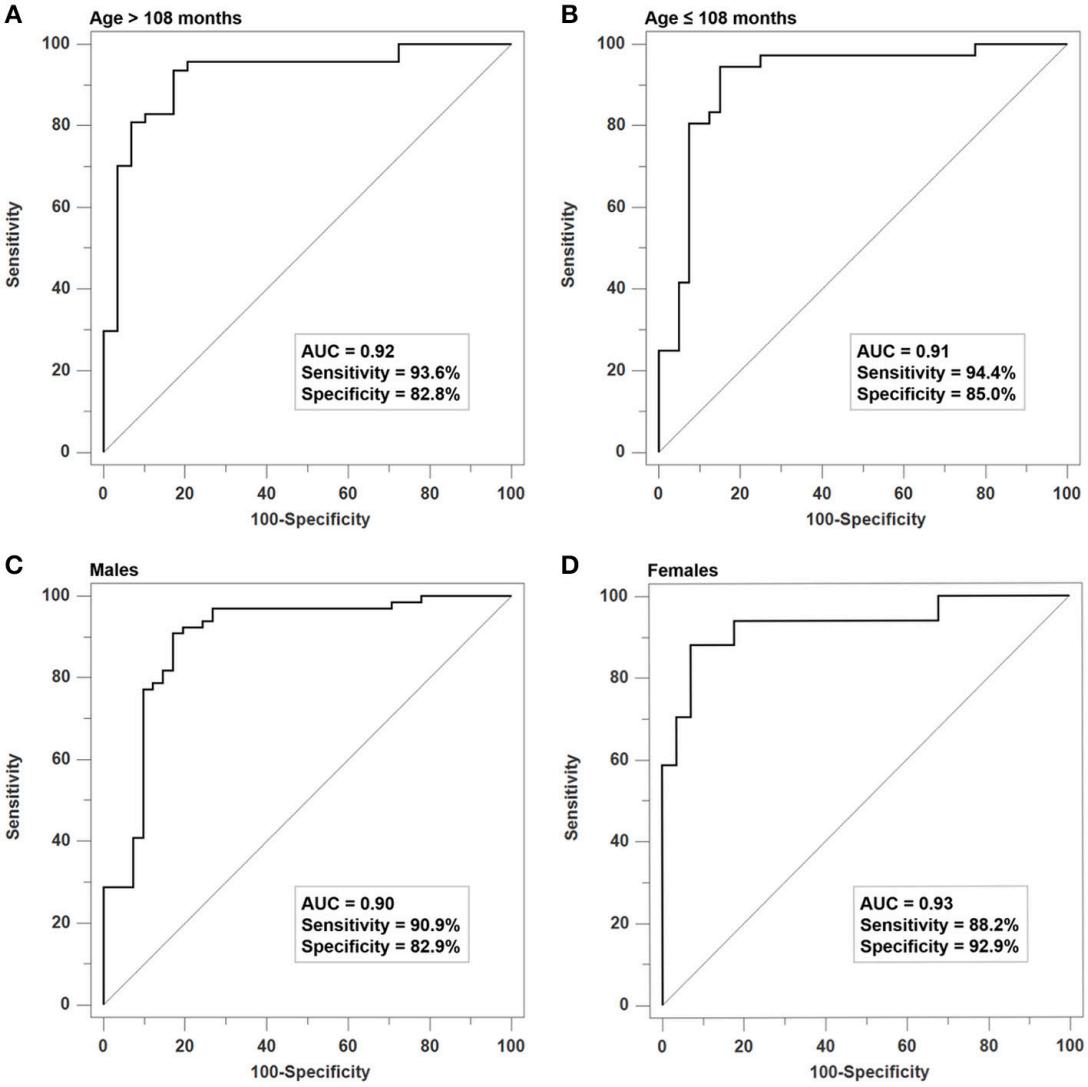
Sensitivity analyses of the SVM Model for age- and gender-stratified samples. The SVM model has good discriminative validity for differentiating patients from controls in **(A)** the older group (≤ 108 months, AUC: 0.93), **(B)** the younger group (>108 months, AUC: 0.91), **(C)** males (AUC: 0.90), and **(D)** females (AUC: 0.94).

As demonstrated in Figure 3, we found that the SVM model could effectively differentiate ADHD patients from controls in both the older group (Figure 3A, ≤ 108 months, AUC: 0.93, *p* < 0.001) and the younger group (Figure 3B, > 108 months, AUC: 0.91, *p* < 0.001). Moreover, the gender-stratified analysis showed that the SVM model can still significantly differentiate patients and controls both in males (Figure 3C AUC: 0.90, *p* < 0.001) and in females (Figure 3D, AUC: 0.94, *p* < 0.001). Lastly, we observed that the probability score yielded by the SVM was positively correlated to the inattentive symptoms rated by parents (*r* = 0.456, *p* < 0.001), teachers (*r* = 0.478, *p* < 0.001), or clinicians (*r* = 0.635, *p* < 0.001), as well as the hyperactivity/impulsivity symptoms rated by parents (*r* = 0.411, *p* < 0.001), teachers (*r* = 0.404, *p* < 0.001), or clinicians (*r* = 0.608, *p* < 0.001).

### Pathways enrichment

We are curious about the possible regulation roles of these biomarker miRNAs, we analyzed the pathways enriched by the biomarker miRNAs' predicted target genes by downloading their target genes from TargetScan 6.2. Of the nine miRNAs with *p* < 0.05, only let-7g-5p, miR-30e-5p, miR-223-3p, and miR-486-5p have predicted target gene data. We then generated a set of 2,510 non-redundant target genes, followed by pathway enrichment analysis with Partek Genomic Suite. As shown in Supplementary Table 4, we identified 56 significant pathways enriched by 2,510 predicted target genes.

## Discussion

To the best of our knowledge, this report is the first to use global screening technology (NGS) to explore candidate miRNA biomarkers for ADHD. Furthermore, we argue that this study is the foremost in providing evidence that combining differential expression levels of miRNAs in WBC may be helpful to differentiate patients with ADHD from healthy control subjects.

ADHD is a highly genetic neurodevelopmental disorder (12). Previous studies have shown that symptoms and neurocognitive deficits of ADHD patients are related to abnormalities in cortex development and functional and structural connectivity among several brain regions (53). Several peripheral (e.g., norepinephrine, 3-methoxy-4-hydroxyphenylethylene glycol, monoamine oxidase, zinc) (4) and genetic biomarkers (e.g., genes belonging to dopaminergic, circadian rhythms and neurodevelopmental systems) (54) have been associated with ADHD both in diagnosis and in treatment response. miRNAs participate in modulating gene expression, resulting in the influence on the transcription of hereditary messages (18), and miRNAs are particularly associated with the abnormal differentiation and maldevelopment of neurons (19, 20). This study used miRNAs in WBC as a circulating biomarker, while miRNAs may be present in exosome within various bodily fluids, including plasma or serum and cerebrospinal fluid (55). However, recent evidence has demonstrated that the interactions between external stimuli and pathological brain processes may be reflected in peripheral tissues, thus facilitating the circulation of miRNA levels as non-invasive and sensitive biomarkers (56). Therefore, miRNA expression in circulation may reflect the genetic network, neuron development, and brain function and may also be correlated with ADHD manifestations (57). Our results support that the expression levels of miRNAs may potentially serve as ADHD biomarkers.

Previous studies have indicated that several miRNAs (miR124-1, miR-598, miR-96, miR-18a-5p, miR-22-3p, miR-24-3p, miR-106b-5p, miR-107, miR-155a-5p, and let-7d) may be associated with ADHD (32, 33, 37, 38). However, none of the aforementioned miRNAs overlapped with those identified in our NGS analyses. The 13 miRNAs that were selected to build the ADHD biomarker panel in the current study have not previously been reported to be involved in ADHD pathophysiology. Several explanations may account for this phenomenon. First, the selection of candidate miRNAs in previous studies (32, 33, 37, 38) relied on underlying neurobiology mechanisms based on literature reviews or the miRNA database. In contrast, we used global screening technology (NGS) to determine the candidate miRNAs, which has a better chance of determining new miRNAs involved in the pathogenesis of ADHD (58). Second, ADHD is a disease with a complex pathophysiology (11, 13). The underlying biological mechanisms of ADHD may not be identical across ethnicities. Finally, all previous studies lacked a validation group to confirm their preliminary results. However, we enrolled an independent validation group (Testing Set) and also verified the findings of the Training Set. Altogether, we suggest that our study methods were considerably more rigorous than previous studies that investigated a similar topic.

We must mention that the patients and controls in our Training Set were not matched in demographic characteristics. Ziats and Rennert (59) reported that the differential expression of miRNAs occurred during the transition from infancy to early childhood. However, we found that the expression of the 13 miRNAs biomarkers identified in our study were not influenced by age, gender, or intelligence quotient (Table 2). Furthermore, our sensitivity analyses obtained a satisfying discrimination validity of the SVM diagnostic panel across various age and gender groups, thus indicating that the differential miRNA expressions were mainly influenced by the disease (ADHD). Moreover, the probability score produced by the SVM model was positively correlated to the ADHD symptom score. In summary, the results reveal that the discriminative validity of the SVM biomarker panel was not influenced by participants' age and sex. However, it is noteworthy that the study herein conduct in an explorative manner. Some miRNAs markers which were selected for establishing the SVM panel only showed borderline statistical significant levels. Moreover, corrections of multiple tests were not performed in this study. The above methodology shortcomings inflated the possibility of false positive results, therefore the findings require further verification in a future study with a larger sample size.

We are curious about the possible regulation roles of the biomarker miRNAs, and we identified 56 significant pathways enriched by 2,510 predicted target genes using Partek Genomic Suite. Of the significant pathways, we are especially interested in axon guidance pathway (*p* = 0.00031), which regulates synaptic transmission and controls neural network development (60). The dysfunction of axon guidance pathways may be correlated with a wide phenotype that involves externalizing mental disorders, including ADHD (61). Therefore, these biomarkers may control the pathogenesis of ADHD through the axon guidance pathway and brain development, and this hypothesis deserves further investigation. To investigate the possible underlying regulation mechanisms of these biomarker miRNAs, we conducted pathway enrichment analysis on the target genes of theses biomarker miRNAs. However, among the significant biomarker miRNAs (*p* < 0.05), only four of them had target gene data, included in pathway enrichment analysis. In other words, more than half miRNAs were missed which was the limitation of this study. In addition, for the miRNAs with *p* < 0.1, we did not have their target gene data so that they were also excluded in target gene enrichment analysis which was another limitation of this study.

Among the initial 2,510 genes, 35 were enriched in Axon Guidance pathway. And, six of them were co-regulated by equal to or more than two miRNAs, including SRGAP3, EPHA3, PLXNC1, RASA1, SEMA3A, and UNC5C. After examining the regulation route of Axon Guidance pathway, we found that these co-regulated genes usually played the critical roles, either the membrane receptor or the signal transduction hub. For example, UNC5C, PLXNC1, and EPHA3 encodes the proteins of membrane receptors responsible for transducing external stimuli into cells. SRGAP3 and SEMA3A function as irreplaceable intermediate of signal transduction. Such multiple-miRNA regulation phenomenon constitutes a sophisticated modulation mechanism.

This study has several methodological issues and limitations that need to be mentioned. First, miRNA-related genetic variations have been reported to alter the expression levels of particular miRNAs (16). Whether genetic polymorphisms in miRNAs affect the protein encoded by the target gene needs to be further investigated. Second, the sample size was small, and ADHD patients and controls in the Training Set were not matched in age and gender. Furthermore, ADHD is generally heterogeneous and can be categorized into different subtypes, and psychiatric comorbidities can be seen in the majority of ADHD patients (62). Due to our small sample size, whether the heterogeneity of ADHD influences the results of miRNA expression was not examined in this study. Third, the specificity of this diagnostic panel needs to be further elucidated in a study that contains patients with neurodevelopmental disorders other than ADHD, such as intellectual disabilities or autism spectrum disorders. Fourth, the effect sizes of the differences in ΔCt values between the ADHD group and control group were small, and corrections of multiple tests were not performed in this study. Thus, the possibility of false positive finding could not be excluded. Fifth, although the exclusion criteria of the participants contained major genetic, metabolic, or infectious diseases. Some minor illnesses, such as nutrition deficiency or minor infectious conditions, may have a marked influence on the WBC transcriptome and affect the results of this study. Finally, the participants in this study were all Han Chinese and recruited from a single site in Taiwan. Further research is necessary to confirm the stability of this biomarker panel and whether our findings can be generalized into various ethnicities or countries.

In conclusion, this study is at the forefront of providing evidence that combining differentially expressed miRNAs can be applied to establish an objective and valid biomarker panel that can differentiate patients with ADHD from healthy control subjects. The miRNA-based biomarkers, as can be measured in peripheral blood, may assist in identifying ADHD in clinical settings. However, the findings of our study require further confirmation with a larger sample size and a cross ethnic validation. Future studies are needed to clarify the gene functions that are modulated by miRNAs and the biological mechanisms that underlie the ADHD pathophysiology.

## Author contributions

L-JW and S-CL participated in study design, patient recruitment, reviewing references, executed the statistical analysis, interpreting data, and drafting the manuscript. L-JW and S-CL are joint first authors and contribute equally to this manuscript. M-JL, M-CC, and W-JC participated in data collection and patient recruitment. S-YL drafted and revised the manuscript. C-WH provided technical and material support. L-HH participated in protocol development. H-CK participated in study design and revised the manuscript. All authors read and approved the final manuscript and contributed to the drafting and revising of the paper.

### Conflict of interest statement

The authors declare that the research was conducted in the absence of any commercial or financial relationships that could be construed as a potential conflict of interest.
