# Supplementary figures and images for: Blood-Bourne MicroRNA Biomarker Evaluation in Attention-Deficit/Hyperactivity Disorder of Han Chinese Individuals: An Exploratory Study
Source: Front Psychiatry. 2018 May 29;9:227. doi: 10.3389/fpsyt.2018.00227 (PMC5987559; doi:10.3389/fpsyt.2018.00227)

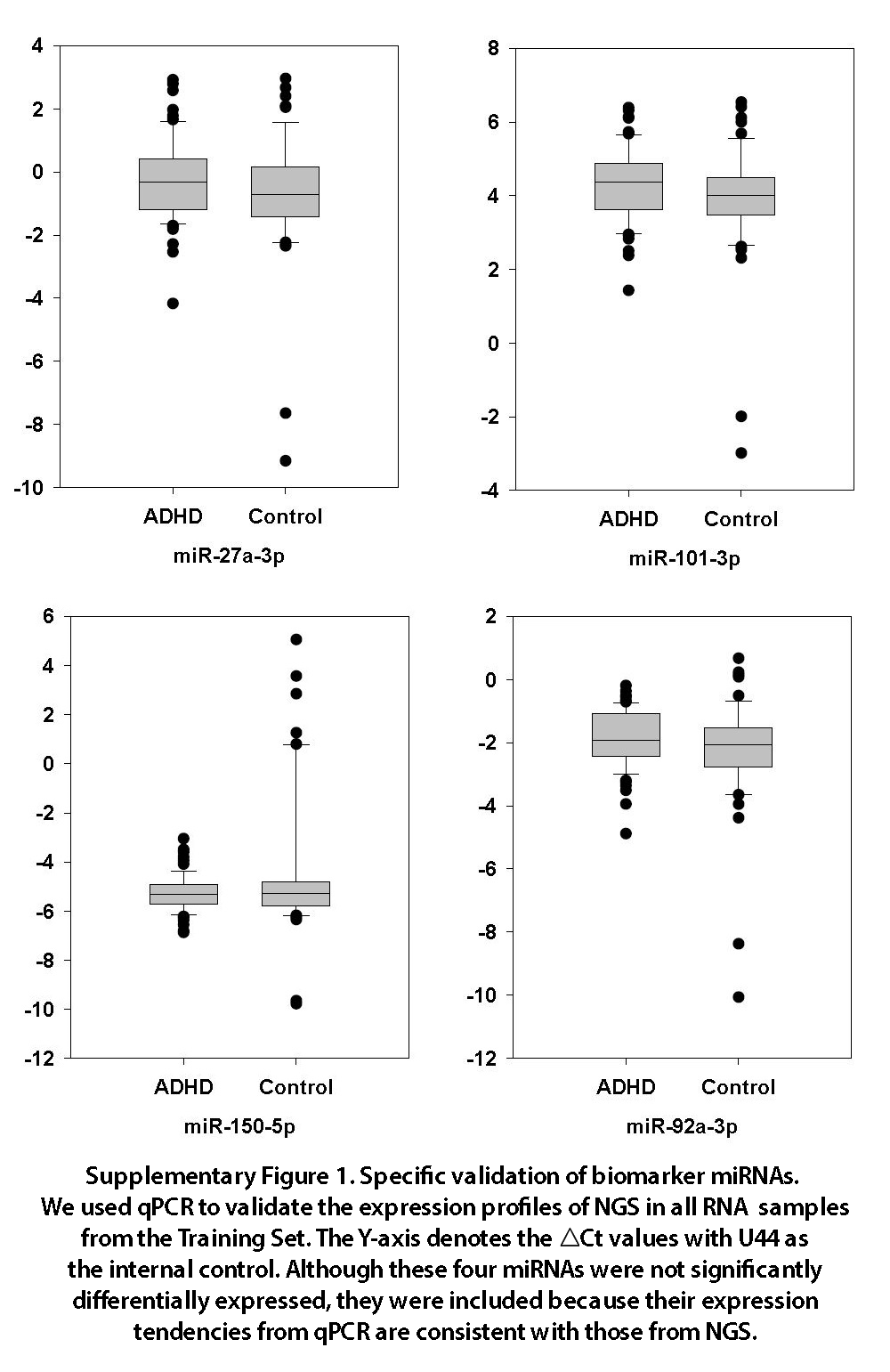

Supplement: Supplementary file 1 [file Image_1.tif]
