# Supplementary material for: Blood-Bourne MicroRNA Biomarker Evaluation in Attention-Deficit/Hyperactivity Disorder of Han Chinese Individuals: An Exploratory Study
Source: Front Psychiatry. 2018 May 29;9:227. doi: 10.3389/fpsyt.2018.00227 (PMC5987559; doi:10.3389/fpsyt.2018.00227)
Supplement: Supplementary file 2 [file Table_1.doc]

**Supplementary Table 1. The TaqMan miRNA qPCR kit.** We used TaqMan miRNA assays to detect miRNA expression profiles. The AB Assay ID and mature sequence for each miRNA are listed.

| **miRNA** | **AB Assay ID** | **Mature MicroRNA Sequence** |
| --- | --- | --- |
| hsa-miR-140-3p | 002234 | UACCACAGGGUAGAACCACGG |
| hsa-miR-27a-3p | 000408 | UUCACAGUGGCUAAGUUCCGC |
| hsa-miR-101-3p | 002253 | UACAGUACUGUGAUAACUGAA |
| hsa-miR-150-5p | 000473 | UCUCCCAACCCUUGUACCAGUG |
| hsa-let7-g-5p | 002282 | UGAGGUAGUAGUUUGUACAGUU |
| hsa-miR-30e-5p | 002223 | UGUAAACAUCCUUGACUGGAAG |
| hsa-miR-223-3p | 002295 | UGUCAGUUUGUCAAAUACCCCA |
| hsa-miR-142-5p | 002248 | CAUAAAGUAGAAAGCACUACU |
| hsa-miR-92a-3p | 000431 | UAUUGCACUUGUCCCGGCCUGU |
| hsa-miR-486-5p | 001278 | UCCUGUACUGAGCUGCCCCGAG |
| hsa-miR-151a-3p | 002254 | CUAGACUGAAGCUCCUUGAGG |
| hsa-miR-151a-5p | 002642 | UCGAGGAGCUCACAGUCUAGU |
| hsa-miR-126-5p | 000451 | CAUUAUUACUUUUGGUACGCG |
