# Supplementary material for: Blood-Bourne MicroRNA Biomarker Evaluation in Attention-Deficit/Hyperactivity Disorder of Han Chinese Individuals: An Exploratory Study
Source: Front Psychiatry. 2018 May 29;9:227. doi: 10.3389/fpsyt.2018.00227 (PMC5987559; doi:10.3389/fpsyt.2018.00227)
Supplement: Supplementary file 3 [file Table_2.doc]

**Supplementary Table 2.** Preliminary results of candidate miRNAs that can serve as biomarkers identified through the NGS technique. Real count denotes transcript per million.

| **ID of miRNA** | **Real Count** | | **Ratios** | |
| --- | --- | --- | --- | --- |
|  | **ADHD** | **Controls** | **ADHD/Controls** | **Controls/ADHD** |
| hsa-miR-29a-3p | 2,857.1 | 1,417.1 | 2.01616 | 0.495992 |
| hsa-miR-142-3p | 3,413.1 | 1,619.1 | 2.108023 | 0.474378 |
| hsa-miR-140-3p | 3,203.1 | 1,855.1 | 1.726645 | 0.579158 |
| hsa-miR-423-3p | 3,962.1 | 1,576.1 | 2.513863 | 0.397794 |
| hsa-miR-192-5p | 5,629.1 | 3,719.1 | 1.513565 | 0.660692 |
| hsa-miR-27a-3p | 5,775.1 | 3,637.1 | 1.587831 | 0.62979 |
| hsa-miR-101-3p | 9,978.2 | 6,370.2 | 1.566387 | 0.638412 |
| hsa-miR-150-5p | 10,905.1 | 6,608.1 | 1.650263 | 0.605964 |
| hsa-let-7g-5p | 17,798.1 | 10,618.1 | 1.676204 | 0.596586 |
| hsa-miR-30e-5p | 17,799.1 | 11,088.1 | 1.605243 | 0.622958 |
| hsa-miR-223-3p | 43,388.1 | 25,353.1 | 1.711353 | 0.584333 |
| hsa-miR-142-5p | 45,635.1 | 29,248.1 | 1.560276 | 0.640912 |
| hsa-miR-143-3p | 51,969.1 | 31,709.1 | 1.638933 | 0.610153 |
| hsa-miR-92a-3p | 64,007.2 | 38,022.2 | 1.683417 | 0.59403 |
| hsa-miR-486-5p | 47,774.2 | 122,205.2 | 0.390934 | 2.557975 |
| hsa-miR-151a-3p | 2,299.1 | 6,025.1 | 0.381587 | 2.620634 |
