# Supplementary material for: Blood-Bourne MicroRNA Biomarker Evaluation in Attention-Deficit/Hyperactivity Disorder of Han Chinese Individuals: An Exploratory Study
Source: Front Psychiatry. 2018 May 29;9:227. doi: 10.3389/fpsyt.2018.00227 (PMC5987559; doi:10.3389/fpsyt.2018.00227)
Supplement: Supplementary file 5 [file Table_4.doc]

**Supplementary Table 4.** The results of pathway enrichment analysis. We downloaded the target genes of let-7g-5p, miR-30e-5p, miR-223-3p, and miR-486-5p from TargetScan 6.2, generating a set of 2,510 non-redundant target genes. Then, we conducted pathway enrichment analysis on the target genes with Partek Genomics Suite. The results are demonstrated as follows.

| **Pathway Name** | **Enrichment p-value** | **Pathway ID** |
| --- | --- | --- |
| FoxO signaling pathway | 8.70E-05 | kegg_pathway_86 |
| Wnt signaling pathway | 1.70E-04 | kegg_pathway_230 |
| PI3K-Akt signaling pathway | 2.73E-04 | kegg_pathway_262 |
| Axon guidance | 3.10E-04 | kegg_pathway_151 |
| Glioma | 4.59E-04 | kegg_pathway_182 |
| Mucin type O-Glycan biosynthesis | 6.03E-04 | kegg_pathway_107 |
| Protein digestion and absorption | 6.27E-04 | kegg_pathway_279 |
| Transcriptional misregulation in cancer | 7.65E-04 | kegg_pathway_34 |
| MAPK signaling pathway | 8.39E-04 | kegg_pathway_261 |
| Non-small cell lung cancer | 1.31E-03 | kegg_pathway_30 |
| Focal adhesion | 1.87E-03 | kegg_pathway_188 |
| Calcium signaling pathway | 1.94E-03 | kegg_pathway_237 |
| ECM-receptor interaction | 2.11E-03 | kegg_pathway_242 |
| Pathways in cancer | 2.35E-03 | kegg_pathway_74 |
| p53 signaling pathway | 2.52E-03 | kegg_pathway_32 |
| Chronic myeloid leukemia | 2.54E-03 | kegg_pathway_69 |
| Ras signaling pathway | 2.57E-03 | kegg_pathway_265 |
| Retrograde endocannabinoid signaling | 3.07E-03 | kegg_pathway_53 |
| Regulation of actin cytoskeleton | 3.59E-03 | kegg_pathway_139 |
| Glutamatergic synapse | 3.67E-03 | kegg_pathway_85 |
| Small cell lung cancer | 3.71E-03 | kegg_pathway_84 |
| Morphine addiction | 4.18E-03 | kegg_pathway_101 |
| Melanoma | 4.66E-03 | kegg_pathway_27 |
| Neurotrophin signaling pathway | 4.75E-03 | kegg_pathway_50 |
| Prostate cancer | 4.82E-03 | kegg_pathway_234 |
| Amyotrophic lateral sclerosis (ALS) | 6.12E-03 | kegg_pathway_25 |
| SNARE interactions in vesicular transport | 7.49E-03 | kegg_pathway_106 |
| Phosphatidylinositol signaling system | 8.32E-03 | kegg_pathway_24 |
| Hippo signaling pathway | 9.18E-03 | kegg_pathway_96 |
| Ubiquitin mediated proteolysis | 1.03E-02 | kegg_pathway_175 |
| GABAergic synapse | 1.05E-02 | kegg_pathway_235 |
| MicroRNAs in cancer | 1.10E-02 | kegg_pathway_281 |
| Pancreatic cancer | 1.12E-02 | kegg_pathway_249 |
| Dilated cardiomyopathy | 1.24E-02 | kegg_pathway_263 |
| Arrhythmogenic right ventricular cardiomyopathy (ARVC) | 1.26E-02 | kegg_pathway_259 |
| Insulin signaling pathway | 1.28E-02 | kegg_pathway_16 |
| HTLV-I infection | 1.33E-02 | kegg_pathway_190 |
| Hypertrophic cardiomyopathy (HCM) | 1.39E-02 | kegg_pathway_62 |
| Glycosaminoglycan biosynthesis - heparan sulfate / heparin | 1.45E-02 | kegg_pathway_80 |
| Circadian entrainment | 1.47E-02 | kegg_pathway_21 |
| Inositol phosphate metabolism | 1.59E-02 | kegg_pathway_102 |
| Long-term potentiation | 1.99E-02 | kegg_pathway_161 |
| Type II diabetes mellitus | 2.01E-02 | kegg_pathway_2 |
| mTOR signaling pathway | 2.15E-02 | kegg_pathway_176 |
| Adrenergic signaling in cardiomyocytes | 2.34E-02 | kegg_pathway_121 |
| Circadian rhythm | 2.36E-02 | kegg_pathway_131 |
